# Deficiency of Splicing Factor 1 (SF1) Reduces Intestinal Polyp Incidence in *Apc^Min/^*^+^ Mice

**DOI:** 10.3390/biology9110398

**Published:** 2020-11-13

**Authors:** Jyotsna D. Godavarthi, Shahrazad Polk, Lisa Nunez, Amruthesh Shivachar, Nancy L. Glenn Griesinger, Angabin Matin

**Affiliations:** 1 Department of Pharmaceutical Sciences, Texas Southern University, Houston, TX 77004, USA; j.godavarthi1424@student.tsu.edu (J.D.G.); s.polk7032@student.tsu.edu (S.P.); linunez@utmb.edu (L.N.); amruthesh.shivachar@tsu.edu (A.S.); 2 Department of Mathematics, Texas Southern University, Houston, TX 77004, USA; Nancy.GlennGriesinger@tsu.edu

**Keywords:** splicing factor 1 (SF1), APC (adenomatous polyposis coli), polyp

## Abstract

**Simple Summary:**

Splicing factor 1 (SF1) is a widely expressed alternative splicing factor that is able to process each piece of genetic information to generate different types of messenger RNAs (or alternate messages). The alternate messages can generate proteins with slightly different structure or function in the cell. Thus, alternative splicing is responsible for the large diversity of proteins that can finely tune cellular function to the cells’ physiological state. Using mouse models for our study, we found that mice expressing reduced SF1 levels develop fewer intestinal polyps. Lowered SF1 levels appear to limit the initiation of polyps. Thus, our studies point to a novel approach for reducing intestinal polyp burden.

**Abstract:**

Background: Splicing factor 1 (SF1) is a conserved alternative splicing factor expressed in many different mammalian cell types. The genetically modified *Sf1+/−* (or *Sf1^β-geo/+^*) mice express reduced levels of SF1 protein in mouse tissues, including in cells of the intestines. Mutational inactivation of human adenomatous polyposis coli (APC) gene deregulates the *Wnt* signaling pathway and is a frequent genetic event in colon cancers. Mice with a point mutation in the *Apc* gene (*Apc^Min/+^*) also develop numerous intestinal polyps at a young age. Our aim was to determine the effect of reduced SF1 levels on polyp development due to the strong driver *Apc^Min/+^* mutation. Methods: We utilized mice genetically deficient for expression of SF1 to assess how SF1 levels affect intestinal tumorigenesis. We crossed *Apc^Min/+^* to *Sf1+/−* mice to generate a cohort of heterozygous mutant *ApcMin/+;Sf1+/−* mice and compared intestinal polyp development in these mice to that in a control cohort of sibling *Apc^Min/+^* mice. We compared total polyp numbers, sizes of polyps and gender differences in polyp numbers between *ApcMin/+;Sf1+/−* and *Apc^Min/+^* mice. Results: Our results showed that *Apc^Min/+^* mice with lower SF1 expression developed 25–30% fewer intestinal polyps compared to their *Apc^Min/+^* siblings with normal SF1 levels. Interestingly, this difference was most significant for females (*ApcMin/+;Sf1+/−* and *Apc^Min/+^* females developed 39 and 55 median number of polyps, respectively). Furthermore, the difference in polyp numbers between *ApcMin/+;Sf1+/−* and *Apc^Min/+^* mice was significant for smaller polyps with a size of 2 mm or less, whereas both groups developed similar numbers of larger polyps. Conclusions: Our results suggest that lower SF1 levels likely inhibit the rate of initiation of polyp development due to *Apc^Min/+^* driver mutation in the mouse intestine. Thus, therapeutic lowering of SF1 levels in the intestine could attenuate intestinal polyp development.

## 1. Introduction

Colorectal cancer (CRC) is the third most common cancer in the US, with the second highest in terms of mortality rates, and with higher incidence and mortality rates in African Americans [1,2]. Although there has been tremendous improvement in screening and detection for this cancer, much still remains to be elucidated regarding genetic susceptibility factors that predispose to this cancer.

Alternative splicing contributes to proteome diversity that is necessary for complex traits especially in mammalian systems [3]. Splicing factor 1 (SF1, also known as mammalian branch point-binding protein (mBBP), zinc finger gene in MEN1 locus (ZFM1), or zinc finger protein 162, ZNF162 or ZFP162) is a ubiquitously expressed and highly conserved splicing factor [4,5]. It is required for early spliceosome assembly and may function as a constitutive splicing factor in lower eukaryotes [6,7] but acts as an alternative splicing (AS) factor in mammalian cells [4,8,9]. SF1 participates in the assembly of the earliest spliceosome complex (E’ complex) during pre-mRNA splicing [10,11,12,13,14,15]. where it interacts with U2 snRNP auxiliary factor (U2AF^65^) to co-operatively bind to the branch point sequence and polypyrimidine tract within the intron of pre-mRNAs [6,16,17,18]. In mammalian cells, SF1 drives alternative splice site choices and can act as a negative or positive regulator of exon inclusion [8,9].

Crosslinking and immunoprecipitation (CLIP) of SF1-bound RNA in HeLa cells found that SF1 targets are mostly mRNAs of protein-coding genes that participate in a great number of cellular pathways, including metabolic pathways, base excision repair, DNA replication and RAS signaling pathways [8,9]. siRNA depletion of SF1 in HeLa cells resulted in changes in the ratio of inclusion or skipping of alternative exons. Moreover, depending on the transcript, the normal ratios of alternatively spliced isoforms were altered or stability of transcripts was affected. Thus, depletion of SF1 alters the expression levels of mRNA variants [8,9].

Splicing is progressively deregulated during normal aging [19,20,21]. Experimental overexpression of SF1 orthologue was found to be sufficient to restore “youthful” splicing patterns and extend lifespan in *C. elegans* [9]. SF1 was found to mediate lifespan extension in *C. elegans* by modulating the metabolic pathway TORC1 components. Interestingly, expression levels of specific splicing factors in some human populations are associated with longevity [21].

Alternative splicing is an important epigenetic mechanism in cellular transformation and cancer development [22,23,24,25,26,27]. Human cancer genome sequencing reveals recurrent, inactivating mutations in splicing factors and their gene targets in a number of malignancies. *SF1* mutations, gene amplifications or deletions are found to occur with highest frequencies in human uterine cancers, melanomas, and stomach and colorectal cancers (cBioportal for Cancer Genomics).

In mice, SF1 is essential during early development. Mice with homozygous deletion of the *Sf1* gene die during embryogenesis [28,29]. Heterozygous *Sf1+/−* mice are viable but have reduced SF1 expression in tissues. Our initial study combined *Sf1+/−* with mouse strains genetically predisposed to develop germ cell derived testicular tumors, where we found that congenital reduction of SF1 significantly decreased testicular tumors [29]. In contrast, it was reported that carcinogen treatment of *Sf1*+/*−* mice increased the number and size of intestinal polyps [28]. We therefore examined how SF1 deficiency affects intestinal adenoma development in mice in the context of germ line mutations in *Apc.*

Mutational inactivation of *APC* is one of the earliest and frequent genetic events in human colon cancers, and the majority of human sporadic colorectal cancers contain adenomatous polyposis coli (APC) mutations [30,31,32]. Germline mutations in APC deregulate the WNT signaling pathway to cause familial adenomatous polyposis (FAP). The (multiple intestinal neoplasia) Min mouse *(Apc^Min/+^*) carries a point mutation in the *Apc* gene and readily develops intestinal polyps [33]. Combining other genetic modifications with *Apc^Min/+^* mice, such as *Dnmt1N*/+, loss of *Trp53* or ER, has been shown to decrease or enhance polyp multiplicity or sizes [34,35,36]. Additionally, genetic modifiers of *Apc^Min/+^* (Mom1 and others) that either enhance or diminish polyp multiplicity or characteristics have been mapped and, in some cases, candidate genes identified [37,38,39].

Our study aimed to determine the genetic consequences of lowered SF1 levels on *Apc^Min/+^*-driven intestinal polyp development. We therefore generated mice heterozygous mutant for both *Sf1 (Sf1+/−)* and *Apc^Min/+^* and compared polyp numbers and sizes between *ApcMin/+;Sf1+/−* and *Apc^Min/+^* mice. Our results show that congenitally reduced SF1 levels resulted in reduction of intestinal polyp development in *Apc^Min/+^* mice. Thus, SF1 levels in intestinal cells influence cellular transformation and polyp development.

## 2. Materials and Methods

### 2.1. Mouse Strains

Animal studies were approved by Texas Southern University Institutional Animal Care and Use committee (IACUC) protocol number 9088. *Apc^Min/+^* (002020: C57BL/6J-Apc<Min>/J) mice were purchased from JAX Labs (Bar Harbor, ME, USA). *Sf1+/−* mice (*Sf1^β-geo/+^*), generated in our lab, were maintained by crossing to 129/Sv [29].

### 2.2. Generation of ApcMin/+;Sf1+/− Mice

*Apc^Min/+^* males were crossed to *Sf1+/−* female mice, and progeny were screened by PCR genotyping. *Apc^Min/+^* mice were on a B6 strain background, and *Sf1+/−* was maintained on a 129 mouse strain genetic background. Thus, all progeny derived from the cross between *Apc^Min/+^* male and *Sf1+/−* female mice were on a mixed B6/129 genetic background. v1F/v1R and gt1F/gt4R primers [29] were used to identify progeny carrying the *Sf1* heterozygous mutant allele. Wild-type and Min mutant Apc alleles were detected by genotyping using a 3-primer PCR reaction, with primers APC1 (wild-type) (GCCATCCCTTCACGTTAG), common antisense APC2 (TTCCACTTTGGCATAAGGC) and (Min mutant) APC3 (TTCTGAGAAAGACAGAAGTTA) [40]. PCR-amplified DNA samples were loaded onto 1% agarose (GoldBio, St Louis, MO, USA) gel for visualization of products using a BIO-RAD ChemiDoc touch imaging system (version 1.2.0.12) (Bio-Rad, Hercules, CA, USA). Siblings positive for *Apc^Min/+^* and *ApcMin/+;Sf1+/−* were allowed to age for at least 4 months, with most being between 4–6 months old before sacrifice to assess for intestinal polyps. Another sibling cohort of *Sf1+/−* and ++ mice, also derived from the same *Apc^Min/+^* and *Sf1+/−* parents, was similarly aged prior to assessing polyp development.

### 2.3. Assessment of Intestinal Polyp Numbers and Size

For each mouse, the intestinal region stretching immediately distal to the stomach until the caecum (termed SI) was dissected out from the peritoneal cavity [41]. Additionally, the colonic region, immediately distal to the caecum stretching to the anus (termed LI), was isolated. The intestine and colon were repeatedly flushed with PBS. SI was divided into approximately 4 equal sections: SI-1, SI-2, SI-3 and SI-4. One side of the intestine/colon tube was snipped opened to visualize the inner surface. The sections SI-1 to SI-4 and L1 were examined under a Leica S9i Stereomicroscope integrated with a digital color camera with a CMOS sensor and an HDMI monitor (Leica Biosystems, Wetzlar, Germany). Starting from the top of SI-1 and systematically moving downwards, the inner surface of each intestinal and colon segment was scanned under the microscope to count polyps and measure the polyp sizes. The majority of polyps were between 1 mm to 5 mm size. Polyps less than 1 mm or greater than 5 mm were included in the 1mm and 5 mm category. Two individuals participated in identifying and counting the polyps. The count data collected from all the mice were collated according to size of polyps and gender for statistical analysis. The intestinal tissues were subsequently stored in formalin.

### 2.4. Immunoblotting

Protein lysates were prepared in radioimmunoprecipitation (RIPA) lysis buffer (sc-24948, Santa Cruz Biotechnology, Inc., Dallas, TX, USA) containing protease inhibitor cocktail and sodium orthovanadate. The amount of protein in the samples was detected using BIO-RAD protein assay according to manufacturer’s instructions (Bio-Rad, Hercules, CA, USA). Proteins were separated by 12% SDS-PAGE gel and blotted onto Immobilon-P membranes (Millipore-Sigma, Burlington, MA, USA). After overnight blocking with 5% non-fat milk, the blots were incubated overnight with the primary antibodies at a concentration of 1/1000: Anti-SF1 (ab223256, Abcam, Cambridge, UK) or GAPDH (sc-365062, Santa Cruz Biotechnology, Inc., Dallas, TX, USA) at 4 °C. The target proteins were detected with the relevant horseradish peroxidase-conjugated anti-human IgG antibody and ECL Western blotting detection reagents (Clarity Western ECL substrate, Bio-Rad, Hercules, CA, USA) and visualized using ChemiDoc touch imaging system, version 1.2.0.12) (Bio-Rad, Hercules, CA, USA) (Figure S1).

### 2.5. Immunohistochemistry

Immunohistochemistry was performed using anti-SF1 antibody (ab223256, Abcam, Cambridge, UK) at a concentration of 1/4000. Heat-mediated antigen retrieval with Tris/EDTA buffer pH 9.0 was performed before staining using a DAB Substrate Kit and peroxidase (HRP) with nickel, (3,3′-diaminobenzidine) (Vectorlabs, Burlingame, CA, USA).

### 2.6. Statistical Analysis

Data collected were sorted according to genotype, gender, number of polyps and size of polyps. Frequency distribution analysis found that data regarding polyp numbers and sizes were not normally distributed. Therefore, we used a nonparametric alternative statistical methodology to the *t*-test, the Mann–Whitney test. The Mann–Whitney test [42] does not make assumptions about the distribution of the data. Instead of testing the difference between means, the Mann–Whitney hypothesis tests for the difference between two medians. Both descriptive and inferential statistical methodologies were performed using the statistical software IBM SPSS (IBM, Armonk, NY, USA). Nonparametric statistical analyses was performed to determine if the total number of polyps differ between groups, for example, between *Apc^Min/+^* (A) and *ApcMin/+;Sf1+/−* (AS) mice. Subsequently, we determined whether polyp sizes (1 mm to 5 mm) were statistically different between *Apc^Min/+^* (A) and *ApcMin/+;Sf1+/−* (AS) mice and, further, if polyp numbers differed between males and females. Similar statistical analyses were used to determine whether polyp numbers differed between *Sf1+/−* and *Sf1*+/+ (wild-type) mice and whether polyp numbers differed between *Apc^Min/+^* (A) or *ApcMin/+;Sf1+/−* (AS) mice and the control population (combination of *Sf1+/−* and *Sf1*+/+ mice).

## 3. Results

### 3.1. SF1 Expression in Nuclei of Intestinal Cells and Polyps

**Figure 1:**
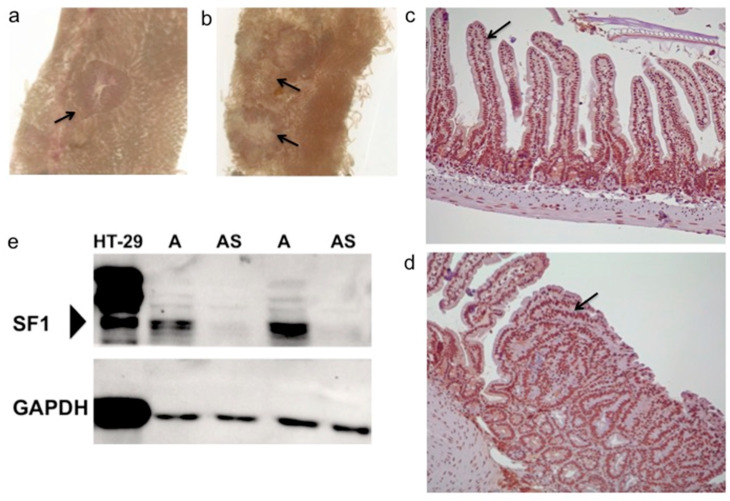
SF1 expression in the intestine. (**a**,**b**) Representative polyps (arrow) in the small intestine of 4-month old *ApcMin/+;Sf1+/−* mice as viewed under the stereomicroscope. (**c**) Immunohistochemistry using anti-SF1 antibody of normal mouse intestine. Arrow indicates splicing factor 1 (SF1) expression in nuclei of intestinal villi and in (**d**) nuclei of adenoma cells of *Apc^Min/+^* mice. (**e**) Immunoblotting using anti-SF1 antibody on cell lysate from HT-29 human colon cancer cells and lysates from *Apc^Min/+^* (A) and *ApcMin/+;Sf1+/−* (AS) mouse intestines. (Bottom) GAPDH expression in the samples.

**Figure 2:**
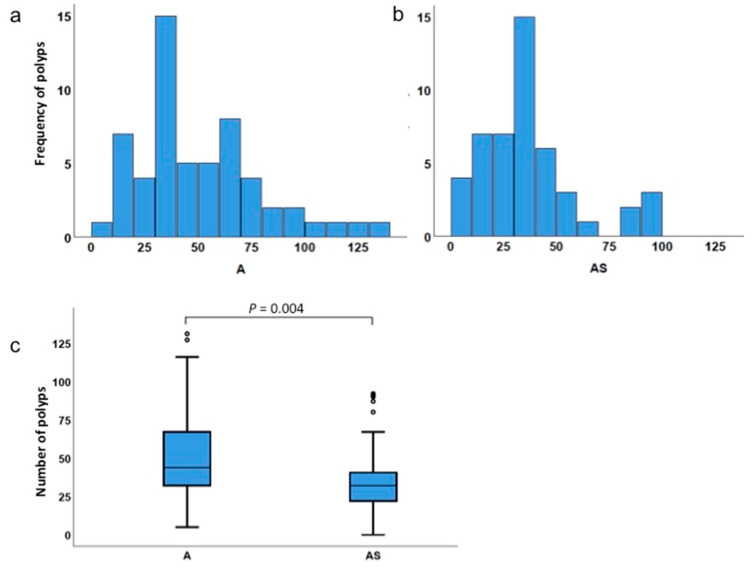
Polyp incidence in *Apc^Min/+^* and *ApcMin/+;Sf1+/−* intestines. (**a**,**b**) Frequency distribution of number of polyps in *Apc^Min/+^* (A) and *ApcMin/+;Sf1+/−* (AS) mice. (**c**) Box plot comparing total number of polyps in *Apc^Min/+^* (A) and *ApcMin/+;Sf1+/−* (AS) mice. The box plots are a graphical representation of the five-number-summary for the number of polyps: the minimum value (the bottom whisker), first quartile (lowest line of the box), median or second quartile (line inside the box), third quartile (top line of the box), and maximum value (top whisker). Actual values are listed in Table 4.

*ApcMin/+;Sf1+/−* and *Apc^Min/+^* mice developed intestinal polyps by 4 months, and the majority of the polyps were of sizes between 1 mm to 5 mm. Polyps were easily visible on the inner intestinal surface, without staining, under a dissecting microscope (Figure 1a,b). The variation in size and number of polyps could be due to the age range, 4–6 months, of mice used for the study (Figure 2a,b). Intestines of mice in control cohorts, wild-type (*Sf1*+/+) and *Sf1+/−* mice, were similarly examined.

Immunohistochemical staining for SF1 in intestinal sections of wild-type B6 mice localized SF1 to the nuclei of epithelial cells of the intestinal villi and crypts (Figure 1c). SF1 was also detected in the nuclei of cells in the intestinal polyps from *Apc^Min/+^* mice (Figure 1d). Further, immunoblotting was used to compare SF1 levels in *ApcMin/+;Sf1+/−* and *Apc^Min/+^* mice. SF1 was significantly reduced in intestinal extracts of *ApcMin/+;Sf1+/−* compared to their *Apc^Min/+^* siblings (Figure 1e). In comparison, very high levels of SF1 were detected in the HT-29 human colon cancer cell line (Figure 1e). Multiple isoforms of SF1, with higher molecular weights, appear to be present in the HT-29 cell line, as detected using the recombinant, monoclonal anti-SF1 antibody (abcam ab223256). Corresponding bands of higher molecular weight isoforms of SF1 were also detected in mouse intestinal lysates, although their expression levels appear to be much lower.

### 3.2. Haploinsufficiency of Sf1 Reduces Intestinal Polyp Formation

*Sf1+/−* was crossed to *Apc^Min/+^* mice, and F1 generation progeny were genotyped to select the four cohorts, heterozygous mutant *ApcMin/+;Sf1+/−* mice, *Apc^Min/+^* mice and controls of genotypes *Sf1+/−* and wild-type (*Sf1*+/+) siblings. Table 1 summarizes the number of *Apc^Min/+^* (*n* = 57), *ApcMin/+;Sf1+/−* (*n* = 48) and control mice (total *n* = 39) examined. Mice were aged for 4–6 months before examination of intestinal polyps in the small and large intestines (SI-1, SI-2, SI-3, SI-4 and L1 sections, as described in the Methods). The inner surface of each intestinal segment was systematically scanned under the microscope to count the polyps and measure each polyp size. Overall, the greatest number of polyps was detected in the SI-3 segment. Most polyps were between 1 mm to 5 mm in size, with the 2 mm-sized polyps being the most numerous, as described below.

Histograms indicating the frequency distribution of the number of polyps in the 57 *Apc^Min/+^* mice and 48 *ApcMin/+;Sf1+/−* mice are shown (Figure 2a,b). The lowest and highest numbers of polyps counted in *Apc^Min/+^* mice were 5 and 131 respectively, and 0 and 92 respectively in *ApcMin/+;Sf1+/−* mice (Table 2). The larger range for *Apc^Min/+^* indicates that the number of polyps in *Apc^Min/+^* mice was more variable than for *Apc^Min/+^;Sf1+/−* mice.

The Mann–Whitney test was used to compare the differences between median polyp numbers in the two independent groups, namely, *Apc^Min/+^* and *ApcMin/+;Sf1+/−* mice, because the variables (polyp numbers) were found to not be normally distributed. Mann–Whitney analysis, which determines if two distributions are equal or not, found that the total number of intestinal polyps in *ApcMin/+;Sf1+/−* (AS) mice was significantly lower compared to that in the *Apc^Min/+^* (A) sibling cohort (*p* = 0.004) (Figure 2c and Table 2). The median numbers of polyps for the *Apc^Min/+^* and *ApcMin/+;Sf1+/−* cohorts were 44 and 32, respectively. (The minimum, first quartile, median, third quartile and maximum values of each box plot are summarized in Table 4). Thus, we conclude that there is a statistically significant difference between the number of polyps in *Apc^Min/+^* mice versus *Apc^Min/+^;Sf1+/−.*

**Figure 3:**
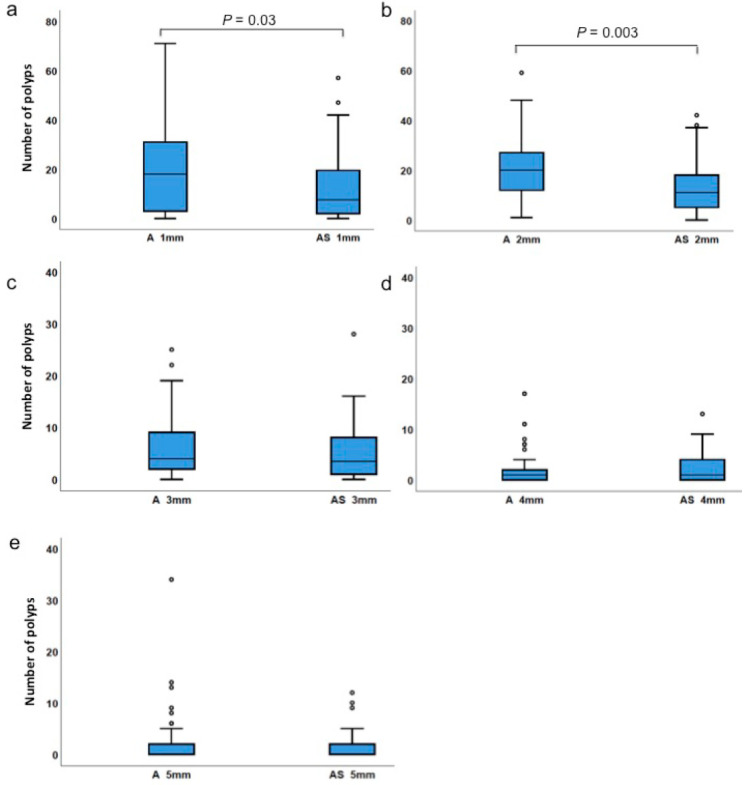
Polyp size comparison. (**a**) Comparison of polyps in *Apc^Min/+^* (A) and *ApcMin/+;Sf1+/−* (AS) mice of sizes of 1 mm, (**b**) 2 mm, (**c**) 3 mm, (**d**) 4 mm and (**e**) 5 mm. Quartile values are represented as described in the legend of Figure 2. Actual values are listed in Table 4.

We then compared how polyp sizes (1 mm to 5 mm) differed between the two groups (Figure 3 and Table 2). When polyps of sizes 1 mm and 2 mm were compared, fewer polyps were detected in *ApcMin/+;Sf1+/−* than in *Apc^Min/+^*. Median values for the number of 1 mm-sized polyps were 18 and 7.5 (*p* = 0.029), and the medians for 2 mm-sized polyps were 20 and 11 (*p* = 0.003) in *Apc^Min/+^* and *ApcMin/+;Sf1+/−* mice, respectively. However, the numbers of polyps with a size of 3–5 mm sizes were not significantly different in both cohorts (Figure 3 and Table 2). Thus, our data showed that congenital lowering of SF1 expression levels led to reduced development of the smaller sized polyps. Because the 1 mm- and 2 mm-sized polyps were more numerous, lowering the numbers of 1 mm- and 2 mm-sized polyps contributed to overall reduced incidence of polyps in *ApcMin/+;Sf1+/−* mice. Because the numbers of 3mm-sized polyps or larger were of similar counts in the two cohorts, this suggests that further growth and enlargement of polyps are independent of SF1 levels.

### 3.3. Low Incidence of Polyps in Sf1+/− Mice

**Figure 4:**
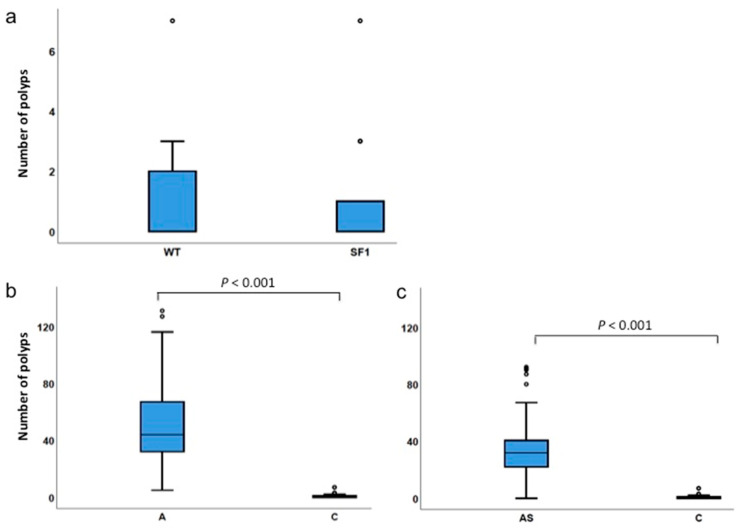
Polyps in WT and *Sf1+/−* mice. (**a**) Comparison of polyp numbers in wild-type (WT) *Sf1+/+* and *Sf1+/−* genotypes; (**b**) *Apc^Min/+^* and control (*Sf1+/+* and *Sf1+/−*) genotypes; and (**c**) *Apc^Min/+^;Sf1+/−* and control genotypes. Quartile values are represented as described in the legend of Figure 2. Actual values are listed in Table 4.

Intestinal polyps of control *Sf1+/−* and wild-type (*Sf1*+/+ or WT) mice were also examined. Statistical analysis did not detect significant differences in polyp numbers between wild-type and *Sf1+/−* mice (Figure 4a). Of the total 39 control mice (*Sf1+/−* and *Sf1*+/+) examined, most developed no polyps, but a few had up to seven polyps/mice (Table 3). Polyp numbers were significantly different between *Apc^Min/+^* and control mice or *ApcMin/+;Sf1+/−* and control cohorts (Figure 4b,c, Table 2).

### 3.4. Gender Differences Affect Polyp Incidences

**Figure 5:**
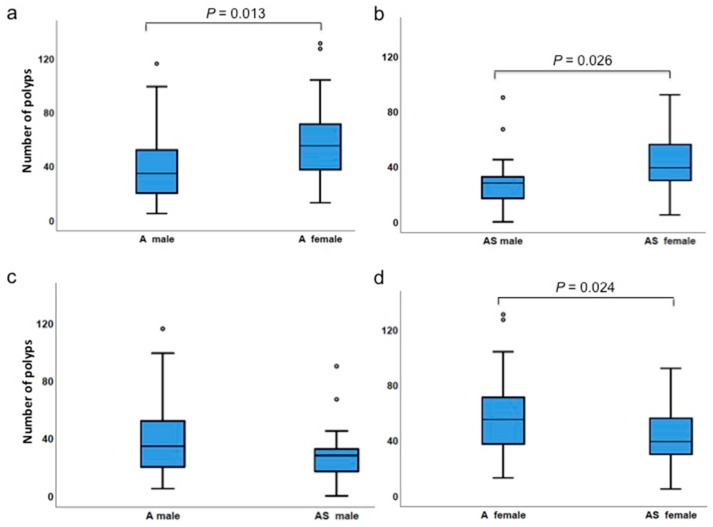
Difference between genders. (**a**) Comparison of polyp numbers in *Apc^Min/+^* (A) males and females; (**b**) *Apc^Min/+^;Sf1+/−* (AS) males and females; (**c**) *Apc^Min/+^* and *Apc^Min/+^;Sf1+/−* males; (**d**) *Apc^Min/+^* and *Apc^Min/+^;Sf1+/−* females. Quartile values are represented as described in the legend of Figure 2. Actual values are listed in Table 4.

Next, we examined whether there were gender differences in polyp development. Interestingly, females of both *Apc^Min/+^* and *ApcMin/+;Sf1+/−* cohorts had higher numbers of polyps compared to males of the same genotypes (*p* = 0.13 for *Apc^Min/+^* and *p* = 0.026 for *ApcMin/+;Sf1+/−*) (Table 2, Figure 5a,b). Polyp numbers in *ApcMin/+;Sf1+/−* females were significantly lower than in *Apc^Min/+^* females (*p* = 0.024; Figure 5d). Although polyp numbers in *ApcMin/+;Sf1+/−* males were lower than in *Apc^Min/+^* males, the results were not statistically significantly different (medians of 34.5 and 28 in *Apc^Min/+^* males and *ApcMin/+;Sf1+/−* males, respectively, *p* = 0.076; Figure 5c).

## 4. Discussion

To date, two genetically modified *Sf1* alleles have been characterized. Shitashige et al. created a gene trap inactivated *Sf1+/−* mouse line, where the trap was inserted in the *Sf1* promoter [28], unlike our *Sf1+/−* mice, where the trap is within the first intron [29]. Notwithstanding this minor technical difference, homozygous *Sf1−/−* mice from the study of Shitashige et al. died due to embryonic lethality, and *Sf1+/−* mice expressed decreased SF1 in their tissues, similar to that observed in our strain. Shitashige et al. reported that treatment of *Sf1+/−* mice with the organotropic carcinogen, azoxymethane (AOM), resulted in a higher number and increased intestinal polyp volume compared to that in wild-type mice [28]. They compared AOM treatment of 4–6 *Sf1+/−* mice to 7–13 wild-type, +/+ mice.

To resolve the question of whether SF1 levels are important for genetic predisposition to intestinal tumorigenesis, we examined a larger cohort of 22 *Sf1+/−* mice and found that most *Sf1+/−* mice developed no polyps, whereas a few developed between one to seven polyps (Table 2 and Table 3). Thus, both *Sf1+/−* and WT (*Sf1+/+*) mice developed few polyps, there was no significant difference between the strains (Figure 4a), and we conclude that congenitally reduced SF1 levels does not predispose to intestinal tumorigenesis.

We then tested how lowered SF1 would affect colon tumorigenesis in genetically predisposed *Apc^Min/+^* mice. Our studies found that the number of intestinal polyps in *Apc^Min/+^;Sf1+/−* mice was significantly lower compared to that in the *Apc^Min/+^* sibling cohort, indicating that lowered SF1 can ameliorate intestinal polyp development. Thus, one possibility regarding the previously reported azoxymethane (AOM)-induced increase in polyp development in *Sf1+/−* mice [28] could be due to greater propensity of AOM-induced driver mutations to persist in intestinal epithelial cells of SF1-deficient mice. Another possibility is that alternatively spliced variants of drug-metabolizing enzymes in SF1-deficient mice may alter the response of intestinal tissues to azoxymethane, thus increasing polyp development in *Sf1+/−* mice.

Reduced SF1 expression in the intestines of *Apc^Min/+^;Sf1+/−* (Figure 1e) was consistent with previous studies where haploinsufficient expression of SF1 was detected in mouse testes from *Sf1+/−* mice [29]. However, our study detected SF1 in nuclei of villi and crypts of the intestine and in *Apc^Min/+^* adenomas (Figure 1c,d). This was different from that reported by Shitashige et al. [28] where they found lower SF1 expression in the crypts and adenomas and correlated SF1 expression with differentiation status of intestinal cells. In contrast, our study found uniform expression of SF1 in nuclei of villus, crypts and adenomas. This difference in expression could be due to the antibody type and source used in the studies. The previous study [28] used polyclonal anti-SF1 antibody (Santa Cruz, CA, USA), which is not commercially available anymore. However, the monoclonal anti-SF1 used in our studies from abcam (abcam, Cambridge, UK) showed nuclear staining for SF1 plus the correct size for SF1 in immunoblotting experiments (Figure 1e). One explanation is that the different anti-SF1 antibodies may each detect specific SF1 isoforms, as immunoblotting (Figure 1e) of human and mouse cells indicates the presence of multiple SF1 isoforms. Thus, although our study confirms nuclear expression of SF1, we cannot draw definite conclusions regarding changes in SF1 with differentiation status of intestinal cells.

When polyps with sizes of 2 mm or less were compared, fewer polyps were detected in *Apc^Min/+^;Sf1+/−* compared to that in *Apc^Min/+^* (*p* < 0.003). However, the numbers of larger sized polyps (>2 mm) were not significantly different in both cohorts. One explanation of this observation is that congenital lowering of SF1 expression in intestinal cells results in decreased initiation or generation of new polyps, resulting in the reduced incidence of small polyps observed in *Apc^Min/+^;Sf1+/−* mice.

The molecular mechanism of how lower SF1 levels reduce polyp development remains to be investigated. It is known that the mRNA targets of SF1 include genes in multiple pathways, including the RAS signaling pathways, base excision repair and DNA replication pathways [8,9]. Furthermore, it has been reported that SF1, together with TCF-4 and β-catenin, promotes gene transcription [43]. This could be one mechanism of generating SF1-associated cancer splice variants. Thus, depletion of SF1 in intestinal cells could result in changes in the expression ratios of alternatively spliced isoforms, stability of oncogenic transcripts or changes in transcriptional activity, which reduce production of cancer splice variants. We expect that SF1 reduction in *Apc^Min/+^;Sf1+/−* mice likely reduces production of multiple oncogenic variants in signaling pathway components, thus ameliorating the strong driver effect of *Apc^Min/+^*. Mechanistic studies remain to be performed to determine how SF1 directly or indirectly affects *Apc^Min/^*^+^ or pathway components to influence polyp development.

Interestingly, females of both *Apc^Min/+^;Sf1+/−* and *Apc^Min/+^* cohorts had higher numbers of polyps compared to males. It is known that the genetic background of mouse strains [44,45] and male hormonal levels can influence polyp incidences [46]. The status of hormonal levels has not been examined in *Sf1+/−* male or female mice. Furthermore, we note that both the B6 and 129 strains have a Mom1^S^ allele with an inactivated *Pla2g2a* gene [44]. Because our cohorts had mixed 129/B6 genetic background, our data indicate that SF1 functions independently of the Mom1 modifier to lower polyp numbers in *Apc^Min/+^;Sf1+/−*mice. The mixed 129/B6 genetic background may also be responsible for the wide range of polyp numbers (0 to 131) observed in our cohorts and as to why females of both *Apc^Min/+^;Sf1+/−* and *Apc^Min/+^* cohorts developed higher numbers of intestinal polyps than males. However, lower SF1 levels appears to be more important for lowering polyp incidence in females than in males because polyp numbers in *Apc^Min/+^;Sf1+/−* females were significantly decreased than in *Apc^Min/+^* females. In humans, males have higher colon tumor incidence than females [1]. Thus, it remains to be investigated how mouse genetic background together with changes in hormonal levels due to reduction in SF1 expression and function modulate intestinal tumorigenesis in the genders.

Polyp incidences in *Apc^Min/+^;Sf1+/−* mice was decreased by 25% to 30%. This level of reduction could be dependent on mouse genetic background, and changing the genetic background of *Sf1+/−* mice to that B6 strain background could further increase the level of suppression of polyp multiplicity in *Sf1+/−;Apc^Min/+^* mice.

Our previous study found lower incidence of TGCT (testicular germ cell tumors) in DND1*^Ter^* mutant mice [29] that express reduced SF1. TGCTs initiate development during embryogenesis in males and develop into visible tumors in 1-month-old male DND1*^Ter^* mice. Similarly, intestinal polyp development is initiated in relatively young *Apc^Min/+^* mice [41,45] as polyps are detected by 4 months. Thus, congenitally lowered SF1 reduces tumor initiation in both the DND1*^Ter^* and *Apc^Min/+^* mouse model systems of cancers that occur in young mice. Whether lower SF1 expression is effective in decreasing incidence in other cancer model systems with longer initiation periods remains to be examined.

## 5. Conclusions

In conclusion, our studies provide important genetic evidence that SF1 levels can attenuate intestinal cancer initiation and development due to the strong driver *Apc^Min/+^* mutation. Further studies using human tissues and cells will be important to understand the mechanistic and cellular role of SF1 in tumorigenesis and whether lowering SF1 could be used as a preventative therapeutic measure for patients predisposed to intestinal tumorigenesis.

## Figures and Tables

**Table 1:** Number of mice examined. Mice were siblings derived from parents *Apc^Min/+^* and *Sf1+/−.*

Cohort Genotype	*n*	No. of Males	No. of Females
*Apc^Min/+^*	57	26	31
*ApcMin/+;Sf1+/−*	48	23	25
*Sf1+/−*	22	13	9
WT (*Sf1*+/+)	17	8	9

**Table 2:** Summary of polyp numbers and results from Mann–Whitney analysis.

Type of Comparison	Genotype	Minimum No. of Polyps	Maximum No. of Polyps	Mean No. of Polyps	Median No. of Polyps	*p* Value
Total polyps	*Apc^Min/+^*	5	131	50.7	44.0	0.004
	*Apc^Min/+^;Sf1+/−*	0	92	35.6	32.0	
1 mm-sized polyps	*Apc^Min/+^*	0	71	20.0	18.0	0.029
	*Apc^Min/+^;Sf1+/−*	0	57	12.5	7.5	
2 mm-sized polyps	*Apc^Min/+^*	1	59	20.4	20.0	0.003
	*Apc^Min/+^;Sf1+/−*	0	42	13.7	11.0	
3 mm-sized polyps	*Apc^Min/+^*	0	25	5.9	4.0	0.487
	*Apc^Min/+^;Sf1+/−*	0	28	5.4	3.5	
4 mm-sized polyps	*Apc^Min/+^*	0	17	2.3	1.0	0.448
	*Apc^Min/+^;Sf1+/−*	0	13	2.6	1.0	
5 mm-sized polyps	*Apc^Min/+^*	0	34	2.1	0	0.729
	*Apc^Min/+^;Sf1+/−*	0	12	1.4	0	
Number of polyps	*Apc^Min/+^* female	13	131	58.2	55.0	0.024
	*Apc^Min/+^;Sf1+/−*female	5	92	41.7	39.0	
Number of polyps	*Apc^Min/+^* male	5	116	41.7	34.5	0.076
	*Apc^Min/+^;Sf1+/−*male	0	90	29.0	28.0	
Number of polyps	*Apc^Min/+^* male	5	116	41.7	34.5	0.013
	*Apc^Min/+^* female	13	131	58.2	55.0	
Number of polyps	*Apc^Min/+^;Sf1+/−*male	0	90	29.0	28.0	0.026
	*Apc^Min/+^;Sf1+/−*female	5	92	41.7	39.0	
Number of polyps	WT (*Sf1+/+*)	0	7	1.0	0	0.974
	*Sf1+/−*	0	7	0.9	0	
Number of polyps	*Apc^Min/+^*	5	131	50.7	44.0	0
	^1^ Control	0	7	1.0	0	
Number of polyps	*Apc^Min/+^;Sf1+/−*	0	92	35.6	32.0	0
	^1^ Control	0	7	1.0	0	

^1^ Control includes both *Sf1+/−* and WT (*Sf1+/+*) mice.

**Table 3:** Frequency distribution of polyps in *Sf1+/+* (WT) and *Sf1+/−* mice.

Cohort Genotype	N	No. with 0 Polyps	No. with 1 to 7 Polyps
*Sf1+/−*	22	14	8
WT (*Sf1*+/+)	17	11	6

**Table 4:** Summary of quartile values of box plots. (**a**) Quartile values from Figure 2 and Figure 3. (**b**) Quartile values from Figure 4 and Figure 5.

(**a**)													
**Percentile**	**A**	**AS**	**A 1**	**AS 1**	**A 2**	**AS 2**	**A 3**	**AS 3**	**A 4**	**AS 4**	**A 5**	**AS 5**	
Min	5.0	0	0	0	1	0	0	0	0	0	0	0	
25	31.0	22.0	3.0	2.0	11.5	5.0	2.0	1.0	0	0	0	0	
50	44.0	32.0	18.0	7.5	20.0	11.0	4.0	3.5	1.0	1.0	0	0	
75	67.5	40.8	31.5	19.8	27.5	18.5	9.0	8.0	2.5	4.0	2.0	2.5	
Max	131.0	92.0	71.0	57.0	59.0	42.0	25.0	28.0	17.0	13.0	34.0	12.0	
(**b**)													
**Percentile**	**A F**	**AS F**	**A M**	**AS M**	**A M**	**A F**	**AS M**	**AS F**	**WT**	***Sf1+/-***	**A**	**AS**	**C**
Min	13.0	5	5	0	5	13.0	0	5	0	0	5.0	0	0
25	37.0	30.0	19.8	17.0	19.8	37.0	17.0	30.0	0	0	31.0	22.0	0
50	55.0	39.0	34.5	28.0	34.5	55.0	28.0	39.0	0	0	44.0	32.0	0
75	74.0	56.5	54.8	33.0	54.8	74.0	33.0	56.5	2.0	1.0	67.5	40.8	1.0
Max	131.0	92.0	116.0	90.0	116.0	131.0	90.0	92.0	7.0	7.0	131.0	92.0	7.0

A 1 = A 1 mm polyp, AS 1 = AS 1 mm polyp, etc. A F = A female, A M = A male, etc. WT = *Sf1+/+*; C = WT plus *Sf1+/−.*

## Supplementary Materials

The following are available online at https://www.mdpi.com/2079-7737/9/11/398/s1, Figure S1: Western blot images.
