# Supplementary figures and images for: Deficiency of Splicing Factor 1 (SF1) Reduces Intestinal Polyp Incidence in ApcMin/+ Mice
Source: Biology (Basel). 2020 Nov 13;9(11):398. doi: 10.3390/biology9110398 (PMC7697247; doi:10.3390/biology9110398)

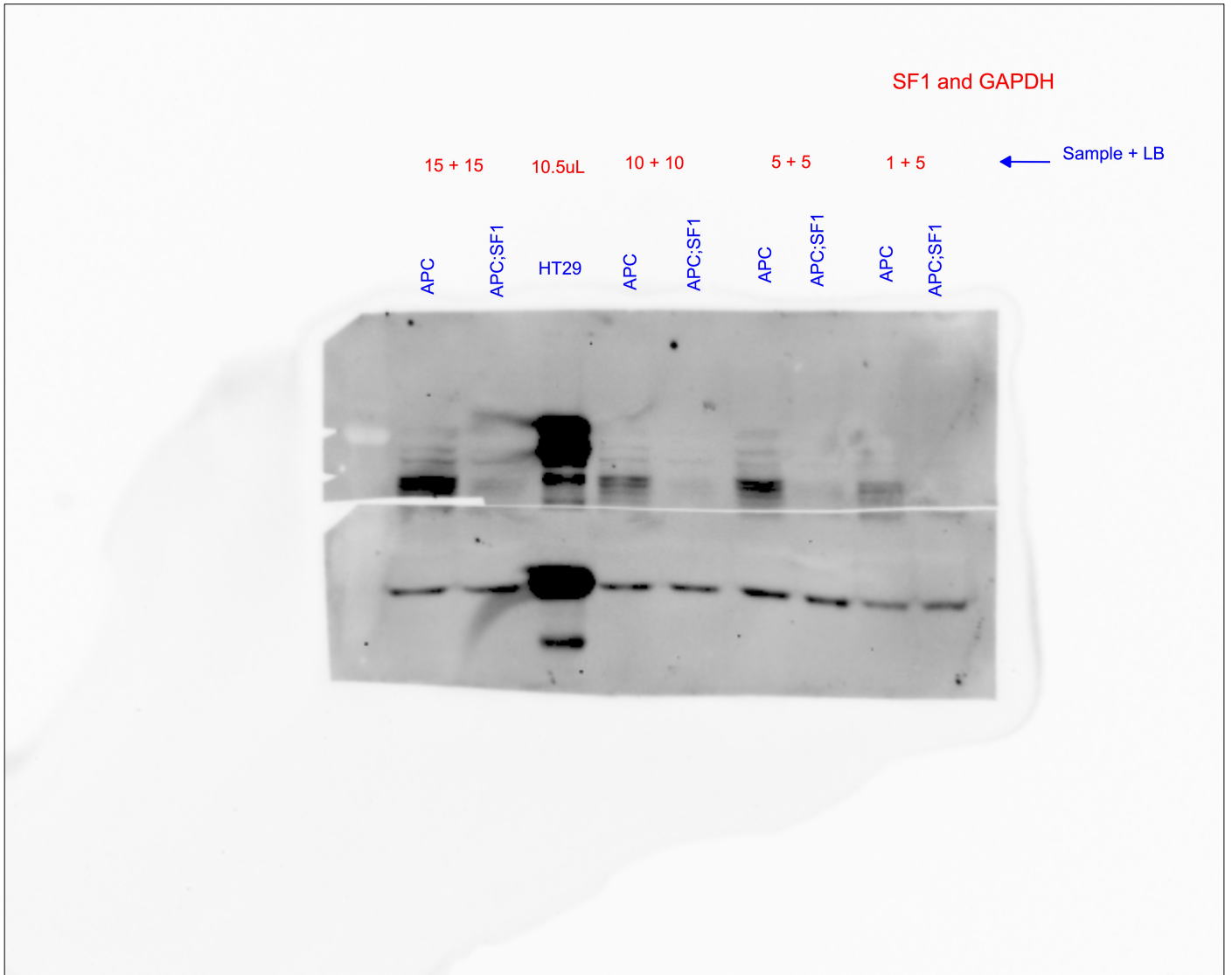

Supplement: Supplementary file 1 [file biology-09-00398-s001.pdf]
